# α-Synuclein is a Novel Microtubule Dynamase

**DOI:** 10.1038/srep33289

**Published:** 2016-09-15

**Authors:** Daniele Cartelli, Alessandro Aliverti, Alberto Barbiroli, Carlo Santambrogio, Enzio M. Ragg, Francesca V.M. Casagrande, Francesca Cantele, Silvia Beltramone, Jacopo Marangon, Carmelita De Gregorio, Vittorio Pandini, Marco Emanuele, Evelina Chieregatti, Stefano Pieraccini, Staffan Holmqvist, Luigi Bubacco, Laurent Roybon, Gianni Pezzoli, Rita Grandori, Isabelle Arnal, Graziella Cappelletti

**Affiliations:** 1 Dept. Biosciences, Università degli Studi di Milano, Milano, Italy; 2 Dept. of Food, Environmental and Nutritional Sciences, Università degli Studi di Milano, Milano, Italy; 3 Dept. Biotecnology and Biosciences, Università degli Studi di Milano-Bicocca, Milano, Italy; 4 Dept. Neuroscience and Brain Technologies, Istituto Italiano di Tecnologia, Genova, Italy; 5 Dept. Chemistry, Università degli Studi di Milano, Milano, Italy; 6 Stem Cell laboratory for CNS Disease Modeling, Wallenberg Neuroscience Center, Department of Experimental Medical Science, Lund University, Lund, Sweden; 7 Strategic Research Area MultiPark and Lund Stem Cell Center, Lund University, Lund, Sweden; 8 Dept. Biology, University of Padova, Padova, Italy; 9 Parkinson Institute, ASST G. Pini-CTO, ex ICP, Milano, Italy; 10 Grenoble Institut des Neurosciences, Grenoble, France; 11 Center of Excellence on Neurodegenerative Diseases, Università degli Studi di Milano, Milano, Italy

## Abstract

α-Synuclein is a presynaptic protein associated to Parkinson’s disease, which is unstructured when free in the cytoplasm and adopts α helical conformation when bound to vesicles. After decades of intense studies, α-Synuclein physiology is still difficult to clear up due to its interaction with multiple partners and its involvement in a *pletora* of neuronal functions. Here, we looked at the remarkably neglected interplay between α-Synuclein and microtubules, which potentially impacts on synaptic functionality. In order to identify the mechanisms underlying these actions, we investigated the interaction between purified α-Synuclein and tubulin. We demonstrated that α-Synuclein binds to microtubules and tubulin α_2_β_2_ tetramer; the latter interaction inducing the formation of helical segment(s) in the α-Synuclein polypeptide. This structural change seems to enable α-Synuclein to promote microtubule nucleation and to enhance microtubule growth rate and catastrophe frequency, both *in vitro* and *in cell*. We also showed that Parkinson’s disease-linked α-Synuclein variants do not undergo tubulin-induced folding and cause tubulin aggregation rather than polymerization. Our data enable us to propose α-Synuclein as a novel, foldable, microtubule-dynamase, which influences microtubule organisation through its binding to tubulin and its regulating effects on microtubule nucleation and dynamics.

Microtubules (MTs) are dynamic polymers consisting of αβ tubulin dimers, which play an essential role in cell shape acquisition and many intracellular processes^1^. In large cells, such as neurons, little is known about how MTs can nucleate in the axonal compartment, far away from the MT-organizing center in the cell body. Further, to date, the regulation of the MT dynamics underlying synaptic functions is still elusive. Many MT-interacting proteins are believed to regulate these phenomena; for example, dispersed γ tubulin complexes are reliable nucleating structures in the axon^2^.

Nowadays, there is increasing evidence for a direct interplay between MTs and α-Synuclein (Syn), a presynaptic unfolded protein widely expressed in brain tissues^3^. Despite the controversial issues on its physiology, Syn has been clearly associated with neurodegeneration. Indeed, Syn overproduction due to multiplications of the *SNCA* locus encoding for Syn and point mutations in the gene itself cause familial forms of Parkinson’s disease (PD)^3^. The underlying pathogenic mechanism is still unclear. Cytotoxicity is currently attributed to Syn oligomers^4^, whose overexpression induces MT disruption in cells^5^. Furthermore, tubulin is known to promote Syn fibrillation *in vitro*^6^, although it is not clear whether destabilization of the MT cytoskeleton potentiates^7^ or prevents^8^ Syn aggregation *in vivo*.

Even though many efforts have been devoted to the identification of a link between tubulin and Syn in pathological contexts, their physiological interaction has been largely ignored. Alim and colleagues^9^ have revealed that wild type (WT) Syn promotes MT assembly, whereas Chen and colleagues have claimed that neither monomeric nor oligomeric Syn influences MT polymerization *in vitro*^10^. Co-immunoprecipitation studies as well as affinity chromatography have also revealed a direct interaction between Syn and free tubulin^11^, but it is still unclear whether Syn forms a complex with tubulin dimers or with higher-order assemblies.

Here we analyse the interaction between Syn and tubulin and we clear up the physiological relevance of the interaction between Syn and MTs. We found that WT Syn undergoes a structural change upon tubulin binding, promoting both microtubule nucleation and dynamics. Furthermore, our data obtained with PD-related Syn mutants provide hints for the pathogenic mechanism in PD.

## Results

### Syn binds to MTs and folds upon interaction with the tubulin α_2_β_2_ tetramer

**Figure 1:**
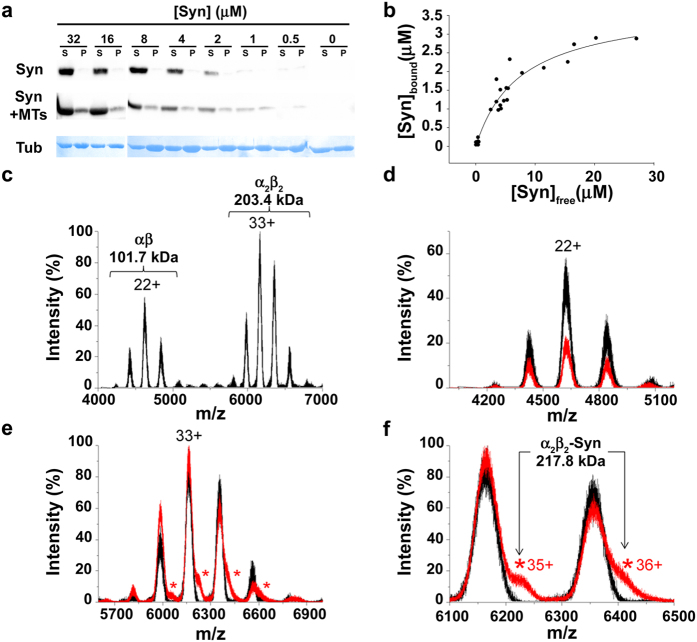
WT Syn interacts with preformed MTs and tubulin α_2_β_2_ tetramer. (**a**) Representative Western blotting of Syn (0–32 μM) recovered in the supernatant (S) or pellet (P) fraction from co-sedimentation assays without (Syn) or after (Syn + MTs) incubation with preformed MTs (Tub) at constant total MT concentration (4 μM tubulin dimers). (**b**) Bound Syn plotted versus free Syn (r^2^ = 0.94). The data reported in the plot derive from at least three different replicates. (**c**) Nano-ESI-MS spectra of 14 μM tubulin: the peak distributions relative to the tubulin dimer and the α_2_β_2_ tetramer are grouped by brackets, with the indication of the measured mass. (**d**) Overlay of spectra of 14 μM tubulin (black), and a mixture of 14 μM tubulin and 14 μM Syn (red), in the *m/z* range 4000–5200 (αβ dimer) or (**e)** in the *m/z* range 5600–7000 (α_2_β_2_ tetramer). The peaks corresponding to the α_2_β_2_/Syn complex are labelled by asterisks. (**f**) Magnification of panel e, in the *m/z* range 6100–6500. The arrows point to the peaks of the α_2_β_2_/Syn complex, labelled by the corresponding charge state. The measured mass of the complex is also indicated. In each panel, the most intense peak of each distribution is labelled by the corresponding charge state.

**Figure 2:**
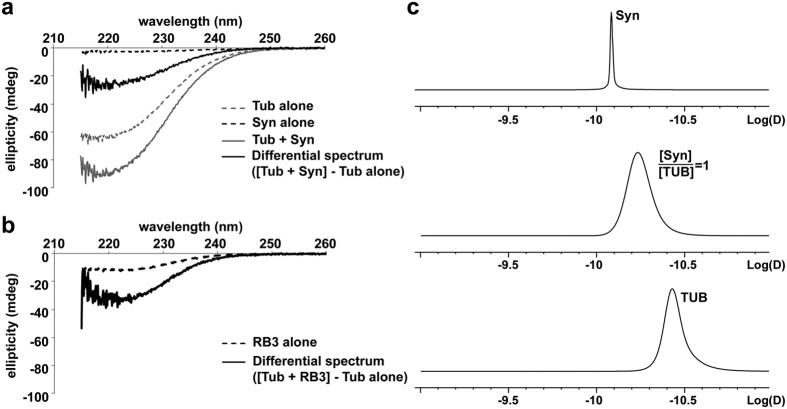
WT Syn interacts with tubulin and acquires secondary structure. (**a**) Far UV CD spectra of isolated 14 μM WT Syn (dashed black line, Syn alone), isolated 14 μM tubulin (dashed grey line, Tub alone), 14 μM WT Syn in the presence of equimolar tubulin (solid grey line, Tub + Syn) and differential spectra (solid black line, differential spectrum, [Tub + Syn] – Tub Alone). (**b**) Far UV CD spectra of isolated RB3-SLD (dashed line, RB3 alone) and differential spectra of 7 μM RB3-SLD in the presence of 14 μM tubulin (solid line, differential spectrum, [Tub + RB3] – Tub Alone). (**c**) ^1^H-NMR diffusion coefficient measurements at 25 °C from 2D-DOSY projections of 27 μM Syn (Syn), Syn/tubulin equimolar mixture ([Syn]/[TUB] = 1) and 27 μM tubulin (TUB).

In order to investigate the mechanism by which Syn regulates MT cytoskeleton and to determine whether Syn interacts with tubulin dimer or with higher-order assemblies, we studied the interaction between purified Syn and tubulin *in vitro*. First of all, we performed co-sedimentation assay (Fig. 1a,b, and Supplementary Fig. S1), an approach which allows investigating the capability of a protein to bind polymerized and stabilized MTs^12^, showing that Syn interacts with preformed MTs (Fig. 1a). The plot of [Syn]_bound_ *versus* [Syn]_free_ (Fig. 1b), which was fitted by nonlinear regression to a standard binding equation, enabled us to calculate an apparent K_d_ of 7.48 ± 1.38 μM, which is indicative of loose binding between Syn and MTs. In addition, differential interference contrast (DIC) and fluorescence microscopy analyses confirmed that WT Syn co-polymerizes with MTs (Supplementary Fig. S2), as revealed by Syn staining along conventional MTs polymerized in the presence of Syn. We also verified the ability of Syn to co-purify with brain tubulin^9^; two purification batches of tubulin were analysed by western blotting using anti-Syn antibodies and resulted to be positive for Syn staining, thus confirming that Syn co-purifies with tubulin (Supplementary Fig. S2). As the tubulin purification involves cycles of polymerization and depolymerization in conditions that should remove the majority of the MT-associated proteins^13^, this result reinforces the idea that Syn can interact also with unpolymerized tubulin^11^. Thus, we investigated the direct interaction between Syn and free tubulin dimers by using native mass spectrometry (MS) and nano-electrospray ionization (nano-ESI). The spectra of 14 μM tubulin and a mixture of 14 μM tubulin and 14 μM Syn (black and red spectra, respectively, in Fig. 1c–f) reveal that Syn forms a specific complex with the tubulin α_2_β_2_ tetramer. The measured mass of this complex (217.8 kDa) was in agreement with the calculated one for a complex of two tubulin dimers (α_2_β_2_) and one Syn molecule. Moreover, the average charge (34.5+) of the complex is close to the expected value for a globular protein of the same mass (36.1+)^14^, suggesting that the complex itself has a compact conformation. However, bound Syn does not necessarily have a globular conformation but it can wrap around the tubulin tetramer in an ordered, but extended conformation. To address this issue, we studied potential conformational changes resulting from tubulin/Syn interaction by far UV circular dichroism (CD). Our data confirmed that Syn is unfolded in the absence of the ligand, whereas an equimolar Syn/tubulin mixture gives an overall secondary structure CD signal, which is more intense than the sum of the signals of the two individual proteins (Fig. 2a). Moreover, the CD signal originating in the mixture shows a gain of signal at 220 nm, which is typical of α-helix structures. Considering that (i) the interaction of tubulin with ligands is widely studied and an increase in its α-helix content has never been observed, and (ii) Syn is a soluble, intrinsically unfolded protein which is able to adopt α-helix structure in adequate conditions^15^,^16^,^17^, we hypothesize that, upon its interaction with tubulin, Syn folds into a structure which is rich in α-helix content. Such a secondary structural transition is reminiscent of the conformational changes associated with the RB3-stathmin like domain (RB3-SLD) upon interaction with tubulin^18^ (Fig. 2b). According to the hypothesis that Syn/tubulin interaction induces α-helical structures in Syn, the CD spectra of Syn complexed with tubulin has been extrapolated (Fig. 2a, solid black line, differential spectrum obtained by subtracting the tubulin spectrum from the one of the complex) and the related α-helix content estimated to 35%. It is worth remembering that such helical content should not reflect the amount of α-helix in a single folded molecule, but rather the average content of α-helix in the whole sample, taking into account both the Syn folded molecules which interact with tubulin and the unfolded ones remaining free in solution. The formation of a Syn/tubulin complex in solution, at neutral pH, has been also investigated by ^1^H-NMR diffusion measurements approach (Fig. 2c). Tubulin alone had a diffusion coefficient (D = 0.37 × 10^−10 ^m^2^sec^−1^) consistent with the presence of αβ dimers in solution, whereas Syn showed a value (D = 0.83 × 10^−10^ m^2^sec^−1^) smaller than the one expected for a globular ~14 kDa protein and consistent with the presence of unfolded species^19^. In the presence of tubulin, the diffusion coefficient measured for Syn significantly decreased from 0.83 × 10^−10^ m^2^sec^−1^ to 0.59 × 10^−10^ m^2^sec^−1^. This clearly indicates the formation of a complex between these proteins. However, since the measured value is actually less than that one expected for a stable 1:1 complex, it should be concluded that, at least in our conditions, a certain fraction of Syn still remains in its free state in solution and that the Syn molecules exchange between the free and bound states at a rate which is fast compared to both the NMR chemical shift and diffusion measurement time scales (fast exchange limit, k_ex_ < 100 sec^−1^). The fraction of free Syn has been estimated within the 45–55% range, leading to a Syn/tubulin dissociation constant between 10 and 20 μM, which is in agreement with the data obtained by the co-sedimentation assay (Fig. 1).

Altogether, our data provide a relatively detailed insight into the binding of Syn to tubulin and MTs, demonstrating, for the first time, the formation of a complex between Syn and the tubulin α_2_β_2_ tetramer, and enabling us to posit a Syn folding step.

### Folded Syn promotes MT nucleation and increases MT dynamics

**Figure 3:**
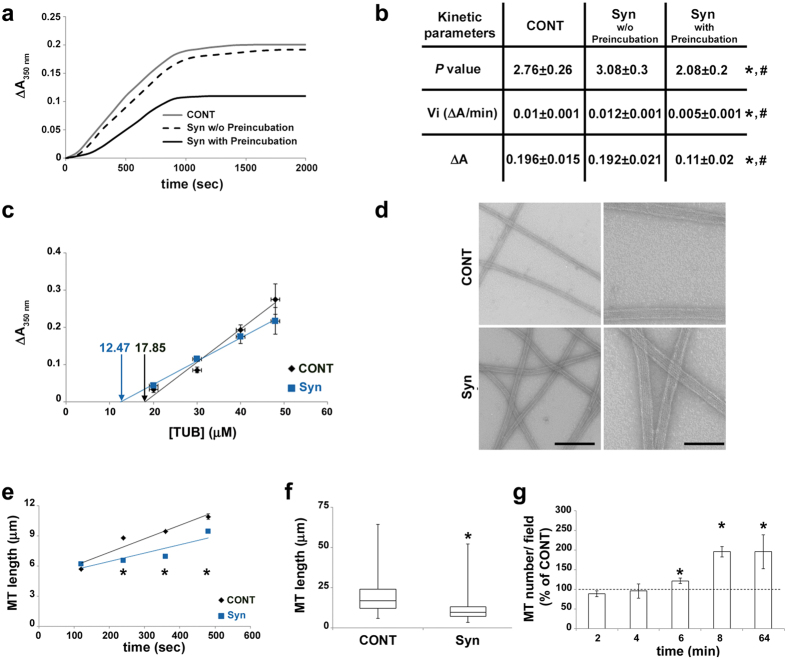
Folded Syn promotes MT nucleation *in vitro*. (**a**) Tubulin assembly was recorded over time by measuring the increase in absorbance variation (ΔA) at 350 nm. Tubulin (40 μM) was polymerized in the absence (CONT) and in the presence of 5 μM of naïve (Syn w/o preincubation) or preincubated (Syn preincubated with tubulin) WT Syn. (**b**) Parameters describing nucleation (*P*, *p = 0.031 vs CONT and ^#^p = 0.002 vs Syn w/o preincubation), initial velocity (V_i_, *p = 0.018 vs CONT and ^#^p = 0.033 vs Syn w/o preincubation) and absorbance variation (ΔA, *p = 0.0016 vs CONT and ^#^p = 0.028 vs Syn w/o preincubation) were calculated from polymerisation kinetics analysis. Values are expressed as mean ± SEM (at least five independent experiments). *p < 0.05 vs CONT, according to ANOVA, Fischer *post hoc* test. (**c**) Final ΔA, obtained at various initial tubulin concentrations, were plotted against tubulin concentration. The tubulin critical concentration (arrows, x-intercept of fitting lines) was calculated in the absence (CONT, black) and in the presence of 5 μM WT Syn (Syn, blue). Plotted values are mean ± SEM (at least three independent experiments). (**d**) Electron microscope images of MTs assembled (40 μM Tubulin) in the absence (CONT) or in the presence of 5 μM WT Syn (Syn). Scale bar, 200 (left) and 100 nm (right). (**e**) MT length measurements during the initial phase of tubulin polymerization (40 μM) in the absence (CONT, black) and in the presence of 5 μM of WT Syn (Syn, blue). Actual p values are 5.1 × 10^−14^ (240 sec), 2.4 × 10^−17^ (360 sec) and 2.3 × 10^−7^ (480 sec). (**f**) MT length measurements in similar conditions at steady state (*p=3.12 × 10^−13^). MT number is at least 600 for each condition, obtained from two independent experiments. *p < 0.05 vs CONT, according to Student’s t-test. (**g**) Quantification over time of the number of MT assembled with WT Syn. Values (mean ± SEM) are expressed as a percentage of the control experiment (without Syn). At least 15 fields obtained from two independent experiments were analysed for each condition (see Supplementary Fig. S2). *p < 0.05 vs CONT, according to Student’s t-test, performed on the raw data. Actual p are 0.024 (6 min), 0.002 (8 min), 0.042 (64 min).

Until now, the effects of Syn on MT polymerization have been studied without pre-incubating Syn and tubulin dimers^9^,^10^. On the other hand, our CD results (Fig. 2) indicate that during the period of the analyses (performed on a time scale of 5–10 min at 20 °C) Syn folds. Thus, we incubated Syn for 10 min at 20 °C with tubulin in order to allow Syn conformational rearrangement prior to carefully analyse tubulin assembly kinetics using a standard tubulin:protein ratio of 8:1 (Fig. 3a,b). We found that Syn molecules that have not been pre-incubated with tubulin did not influence tubulin kinetics. By contrast, pre-incubated Syn strongly impacts MT assembly (Fig. 3a) by reducing by half the initial velocity of polymerization (V_i_) and the MT assembly at the plateau (ΔA), as shown in Fig. 3b. Next, by plotting log(A(t)/A∞) against log(t) we extrapolated the parameter *P*, which is indicative of the successive steps during the nucleation phase^20^, and is significantly decreased compared to tubulin alone (Fig. 3b). The effect of pre-incubated Syn on *P* value and its concomitant ability in significantly reducing tubulin critical concentration (Fig. 3c), underscored the ability of folded Syn to nucleate MTs. We confirmed the presence of MTs in these conditions by electron microscopy and negative staining analysis (Fig. 3d). We next estimated the length and the number of MTs assembled in the presence of folded Syn, which revealed that Syn induces the formation of shorter and more abundant MTs with respect to controls (Fig. 3e–g, see also Supplementary Fig. S3). The significant increase of MT number perfectly agrees with the induction of MT nucleation. However, the significant reduction of the MT mean length also suggests that, besides stimulating MT nucleation, Syn should reduce MT elongation, either by slowing down MT growth rate or increasing catastrophe frequency, as strongly supported by the reduction in the initial velocity of polymerization. Notably, this would explain the decrease in total polymer mass (ΔA in Fig. 3a,b and Supplementary Fig. S3), which is in contrast to the expected effect of a nucleation inducer.

**Figure 4:**
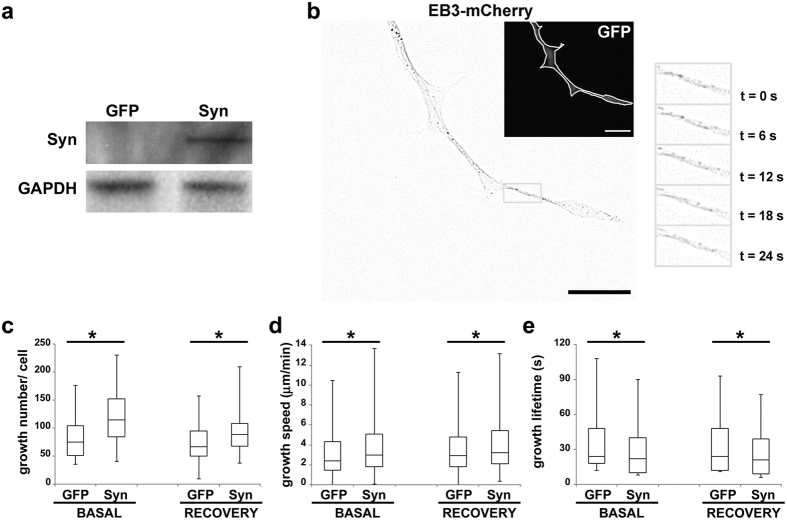
WT Syn increases MT dynamics in neuronal cells. (**a**) Representative western blot showing the levels of expression of human Syn (Syn) in 5 days NGF-differentiated control (GFP) and transfected (Syn) PC12 cells. The antibody used (Sigma-Aldrich) recognized both human and rat Syn. Glyceraldehyde 3-phosphate dehydrogenase (GAPDH) is used as loading reference. (**b**) Representative micrographs of double transfected PC12 cells differentiated 3 days with NGF, showing the EB3 comets (EB3-mCherry, inverted contrast) and, in the inset, the GFP channel (GFP, grey line marks the cell boundary). The grey box indicates the area from which the frames of the time series were taken (elapsed time is shown). Scale bar, 5 μm. Box plots of the number (**c**, *BASAL = 0.007, *RECOVERY = 0.005), speed (**d**, *BASAL = 0.02, *RECOVERY = 0.007) and lifetime (**e**, *BASAL = 0.02, *RECOVERY = 0.02) of MT growths, in each experimental condition. BASAL indicates cell cultures maintained at 37 °C whereas RECOVERY indicates registration during the rewarming phase after 30 min at 4 °C. At least 30 cells were analysed per condition, for a total number of 2200 tracks, or more. *p < 0.05 vs GFP, according to Student’s t-test. The analyses of the relative neurite’s area are reported in Supplementary Fig. S5.

Thus, we used video-enhanced differential interference contrast (VE-DIC) light microscopy to clear up the effects of WT Syn on MT dynamics (Table 1). Tubulin (10 and 15 μM) was assembled from purified axonemes, in the absence or in the presence of increasing concentrations of Syn (0–15 μM). These protein to protein ratios are comparable to those observed *in vivo,* since the actual cellular concentration of tubulin is up to 40 μM^21^ and the estimated presynaptic Syn concentration varies between 30 and 60 μM in neurons^22^. At low tubulin concentration (10 μM), Syn enhanced catastrophe frequency, 4 to 6.5-fold in comparison to the control. A similar, albeit smaller, effect was observed at the highest tubulin concentration (15 μM), with a 3-fold increase in the catastrophe frequency in the presence of Syn compared to tubulin alone. With the two highest Syn concentrations (10 and 15 μM), we also noticed a significant increase in the MT growth rate in the presence of 15 μM tubulin. These results would indicate that Syn increases MT dynamics by speeding up the growth rate and promoting catastrophe events. In order to confirm these data in a neuronal cell line we used differentiated rat PC12 cells, which naturally express rat Syn starting from 7^th^ day of NGF treatment^23^. We transfected PC12 cells with a construct coding for human Syn and treated them with NGF for three days. As shown in Fig. 4a, parental PC12 cells lack of endogenous Syn whereas transfected ones express human WT Syn. First of all, we confirmed the well-known interaction of Syn with βIII-tubulin^5^, either in PC12 cells overexpressing human Syn (Supplementary Fig. S4) or in human neurons derived from embryonic stem cells expressing the endogenous Syn (Supplementary Fig. S5). In addition, we highlighted a high degree of co-localization with tyrosinated α-tubulin, which is associated with the most dynamic MTs. Thus, to directly investigate MT dynamics in cells, we performed live cell imaging taking advantage of end-binding protein 3 (EB3)-mCherry^24^, a fluorescent protein that specifically binds growing MT plus-ends. We visualized MT dynamics under basal conditions (cells kept at 37 °C) or during the MT recovering phase (rewarming at 37 °C), which follows a cold-treatment (30 min at 4 °C), in differentiated PC12 cells co-expressing EB3-mCherry and GFP-Syn or GFP alone (Fig.4). This approach shows that Syn increases the number of detectable growing MTs per neurite (Fig. 4c), even though the analysed surfaces do not change (Supplementary Fig. S6), and thus demonstrates that Syn favours MT (re)nucleation *in cell*. Moreover, Syn accelerates MT growth (Fig. 4d) and reduces MT lifetime (Fig. 4e). The latter observation is consistent with the enhancement of the catastrophe frequency reported by VE-DIC microscopy (Table 1). Altogether, our data demonstrate that, upon interaction with the tubulin α_2_β_2_ tetramer, Syn acquires helical structure and becomes able to govern multiple steps of MT assembly and dynamics, such as nucleation, growth rate and catastrophe frequencies, in a purified system as well as in a neuronal cell model.

### Syn displays sequence similarity to stathmin

**Figure 5:**
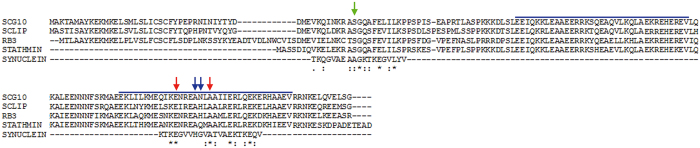
Syn displays sequence similarities with stathmin. Multiple alignment of Syn with four members of stathmin family was performed by ClustalW. Blue lines delimitate the domains of stathmin family involved in tubulin binding. Arrows mark the sites of Syn pathological mutations: Ala30 (green arrow), the conserved Glu46 and Ala53 (red arrows), His50 and Gly51 (blue arrows). Asterisks mark invariant positions, while dots and colons highlight semi-conservative and conservative substitutions, respectively.

Syn displays striking structural and functional similarities with the tubulin-interacting protein stathmin. Both of them are about 14-15 kDa, intrinsically disordered proteins and capable of adopting α-helix conformation upon interaction with binding partners^18^,^25^ (Fig. 2a). It is noteworthy that Syn, like stathmin and RB3-SLD, interacts with the tubulin α_2_β_2_ tetramer and promotes MT catastrophes. Thus, we explored sequence similarities between Syn and the members of the stathmin family. Pairwise alignment of WT Syn to stathmin showed about 20% identical residues and over 50% conservative substitutions. Two possible tubulin-interacting domains have been proposed for Syn but the pathological point mutations, which impair the tubulin binding, are outside the suggested regions^9^,^26^. Therefore, searching for a tubulin-related physiological relevance for the region including the mutations, we decided to align two 21-residue stretches surrounding the residues 30 or 53 in the Syn polypeptide chain (Fig. 5). Interestingly, the fragment centered around Syn residue 53, in which four of the five PD-linked point mutations are clustered^27^, aligned to one of the functionally relevant regions of the stathmin family (Fig. 5, blue lines), namely the tubulin-binding domains^28^. This region displays multiple invariant residues (Fig. 5 asterisks), including the sites of the Syn pathological mutations A53T and E46K (Fig. 5, red arrows), besides several other conservative or semi-conservative substitutions (Fig. 5, colons and dots, respectively). Furthermore, we completed the analysis in order to align the entire region 1–100 of Syn polypeptide chain to the stathmin-family (Supplementary Fig. S7), since we suggest that Syn acquires an α-helical structure (Fig. 2) and this is the domain of Syn that folds upon interaction with vesicle. As the two domains reported in Fig. 5 showed a good conservation, we anchored them and aligned the other stretches of Syn to the remaining regions of the stathmin-family. These regions display a very poor conservation and, thus, this result reinforces the idea that the region around the pathological mutations might be important for binding to tubulin and that its point mutations likely compromise Syn/tubulin interaction. All these data demonstrate that Syn and stathmin share physicochemical and functional properties, and the good alignment of the fragment 43–63 of Syn with a functionally relevant region of stathmins strongly indicates that Syn and proteins belonging to the stathmin-family may be involved in same biological processes, such as the regulation of MT cytoskeleton.

### Pathological Syn mutations corrupt Syn/tubulin interaction

**Figure 6:**
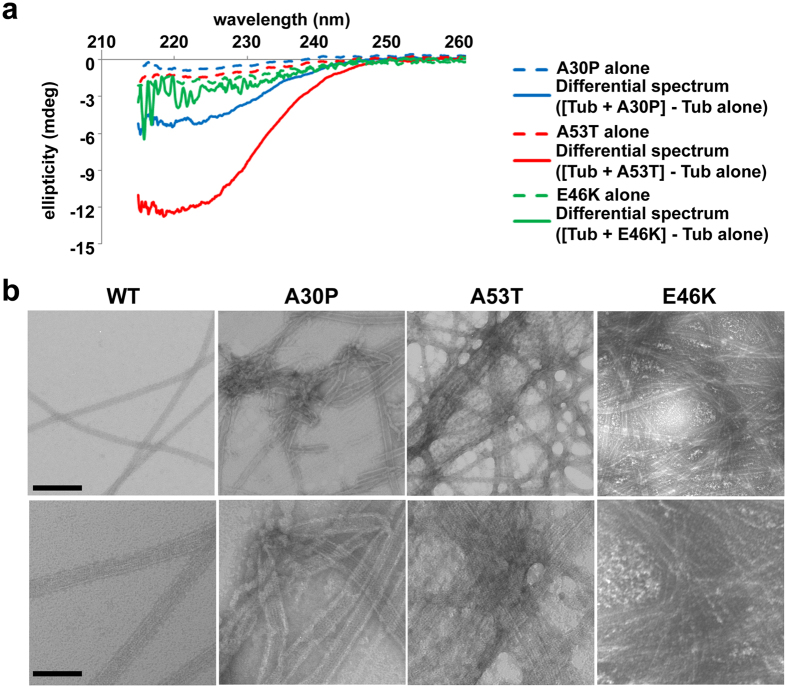
Pathological Syn mutations corrupt Syn/tubulin interaction. (**a**) Far UV CD spectra of 14 μM mutated Syns, A30P (blue lines), A53T (red lines) and E46K (green lines), obtained with naïve proteins (dashed lines, alone) or by the difference between the spectrum of Syns in the presence of equimolar amount of tubulin minus the spectrum of tubulin alone (solid lines, differential spectrum). (**b**) Electron microscope images of MTs assembled *in vitro* (40 μM tubulin) in the presence of 5 μM of WT (WT) or mutated (A30P, A53T and E46K) Syns. Scale bars, 200 (top) and 100 nm (bottom).

Having shown that four out of five PD-linked mutations map to the putative tubulin-binding domain of Syn (Fig. 5), these substitutions are expected to have a profound effect on Syn interaction with tubulin and, reasonably, on tubulin-induced folding of Syn. Indeed, CD analyses revealed that the A53T variant is much less sensitive than WT Syn to the structuring effect of tubulin (Fig. 6a, red lines) whereas the E46K mutant loses almost completely the ability to fold in the presence of tubulin (Fig. 6a, green lines). Although the pathological A30P mutation mapped far away from the putative tubulin-interacting domain we proposed (Fig. 5), this amino acid substitution has the potential to interfere with tubulin-induced folding of Syn, as confirmed by CD analyses (Fig. 6a, blue lines). In order to verify that mutated Syns used in the assays were not aggregated, thus to avoid unwanted effects on tubulin assembly, we analysed them by size exclusion chromatography. We observed a single peak for all the Syn corresponding to a molar mass of 14 kDa (Supplementary Fig. S8), which indicates the presence of monomeric Syn. Moreover, we observed that MTs assembled in the presence of Syn mutants (all pre-incubated with tubulin) are crowded and surrounded by abundant protein aggregates (Fig. 6b and Supplementary Fig. S2), which were not present at the beginning of the experiments (see size exclusion chromatography results, Supplementary Fig. S8) but rather are formed during the incubation. These aggregates should be composed of both tubulin and Syn; indeed, very few and small aggregates are observed both in control conditions, i.e. without adding Syn, meaning that they are made of tubulin, and in the presence of WT Syn (Supplementary Fig. S8). Since aggregates in the presence of mutated Syns (Supplementary Fig. S8) are also recognized by the Syn antibody (Supplementary Fig. S2), they should contain Syn. Interestingly, the aggregation propensity of the mutated Syns seems to be inversely proportional to their ability in folding in the presence of tubulin (Fig. 6a and Supplementary Fig. S8). Therefore, our data reveal that Syn mutants are much less sensitive than WT Syn to tubulin-induced folding and mainly promote tubulin aggregation. This could potentially impair the correct organization of the MT system impacting on the neuronal processes in which MTs and Syn are implicated.

## Discussion

As stated by Feng and Walsh^29^ a decade ago: “Protein-protein interactions are a little like human relationships. Some are dedicated, faithful and lifelong, while other relationships are brief flings with a promiscuous variety of partners that may leave no lasting trace or may induce profound changes”. Here we show that the interplay between Syn and tubulin is a multifaceted protein-protein interaction. Indeed, their encounter triggers the structural rearrangement of Syn which, in turn, regulates the birth, the growth and the lifespan of individual MTs. Therefore, we set forth the hypothesis that Syn is a MT “dynamase”, a term introduced by Erent and colleagues^30^ for Kinesin-8, which is able to regulate both MT nucleation and catastrophes in *S. pombe*, exactly what Syn does, regulating nucleation, growth rate and catastrophe frequency of MTs, *in vitro* and *in cell*.

**Figure 7:**
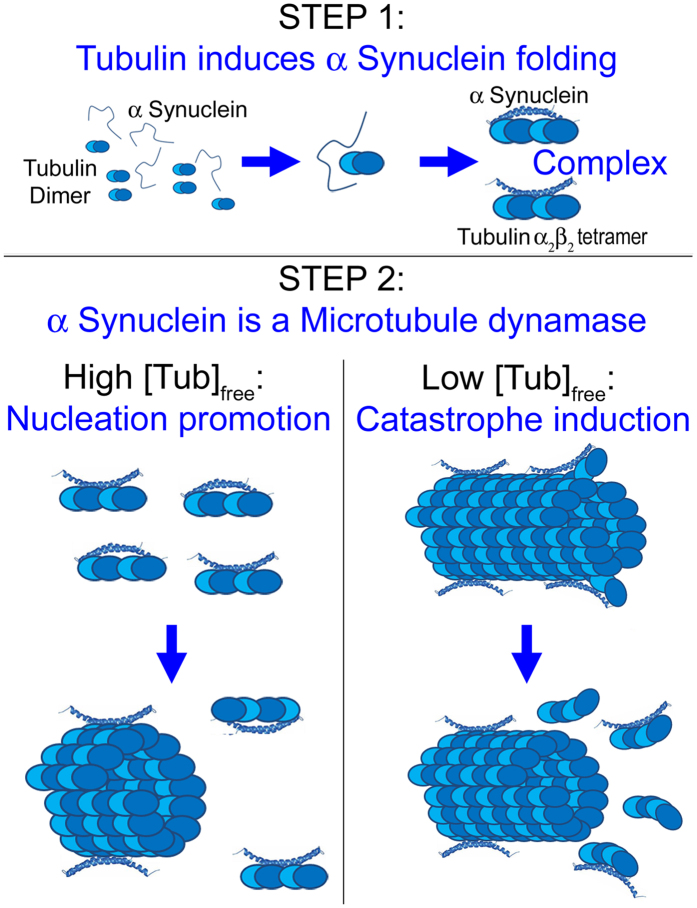
Model for Syn/MTs interaction. Syn senses and binds free tubulin dimers forming a specific complex with tubulin α_2_β_2_ tetramer and acquiring α-helix conformation (STEP 1). Afterwards, Syn behaves like a tubulin deliverer promoting MT nucleation (STEP 2-High [Tub]_free_). When entrapped in the MT wall, Syn promotes MT catastrophes probably inducing changes in intra- or inter-dimer angles (STEP 2-Low [Tub]_free_).

MTs exhibit non-equilibrium dynamics, which depends on free-tubulin concentration. In cell systems, a constant free tubulin concentration, and the presence of a multitude of MT-interacting proteins likely buffers and mitigates perturbations in MT dynamics, conferring a degree of robustness and homeostasis to the MT cytoskeleton^31^. Nevertheless, stressful conditions exist (i.e. neuronal aging) and under these conditions the free tubulin content may considerably change. When tubulin concentration is high, the initial response of a MT-associated protein will be potent and it would induce MT assembly, as we observe at the beginning of assembly kinetics in the presence of Syn (Fig. 3) or in VE-DIC experiments performed at high tubulin concentrations (Table 1). In the mechanistic model we propose, the ability of WT Syn to interact with the tubulin α_2_β_2_ tetramer enables Syn to fold (Fig. 7, STEP 1) and, possibly, to act as a tubulin carrier by delivering small tubulin oligomers (α_2_β_2_ tetramers), which have been recently proposed, although it is controversial, to promote MT nucleation and MT elongation^32^,^33^,^34^. Thus, Syn could either crosslink tubulin heterodimers, inducing nucleation (Fig.7, STEP 2-high [Tub]_free_), and/or stabilize them in a favourable orientation promoting supramolecular interactions involved in MT formation. As net polymerization is promoted, the free tubulin concentration drops down, mitigating the assembly-inducing activity of Syn, as observed at the steady state of assembly kinetics (Fig. 3) or with low tubulin concentrations in VE-DIC experiments (Table 1), when catastrophe stimulation prevails. How are these catastrophe events induced? Two possible mechanisms have been proposed for the catastrophe-promoting activity of stathmin^35^: (i) sequestering of soluble tubulin into assembly-incompetent tubulin/stathmin complexes, which has the same stoichiometry of Syn/tubulin complexes identified in the present study (Fig. 1), or (ii) direct binding of stathmin to MT ends, which would increase the frequency of switching from growth to shortening. It is unlikely that Syn acts by sequestering tubulin dimers, since such a phenomenon would reduce MT growth rate, in contrast to our observations (Table 1). We hypothesize that, once bound to the MT lattice, Syn could affect the whole stability of the polymer by inducing conformational changes in the intra- or inter-dimer angle and thus amplifying the intrinsic tendency of MTs to undergo catastrophes (Fig. 7, STEP 2-low [Tub]_free_). Nevertheless, further studies are needed to definitively solve the mechanism by which Syn promotes MT catastrophes.

Here, we also demonstrated that PD-linked Syn variants do not exhibit α-helical folding upon tubulin binding and induce tubulin aggregation. A30P mutation is located on the short helix of the lipid-folded Syn^27^, and the fact that the polypeptide region including this mutation is not part of the putative tubulin-interacting domain could explain why it is the most “benign” PD point mutation^36^. Conversely, the other 4 described point mutations of Syn (E46K, H50Q, G51D and A53T) fall inside a region corresponding to the tubulin-interacting domain of proteins belonging to the stathmin family. We are aware that this region maps outside the tubulin-binding regions previously proposed for the WT^9^,^26^., which seem to be unique for Syn and do not share homology with other known tubulin-interacting proteins. Nevertheless, the two previously reported binding domains are diverse, sharing only 4 residues and completely excluding the region in which there are the pathological mutations. Alim and colleagues^9^, tried to justify the tubulin-aggregating effect of the A30P and A53T mutants saying that these mutations can alter the Syn folding, exactly what we showed here (Fig. 6). Our results propose an alternative tubulin-binding domain that well matches with the ones of the stathmin-like proteins, and which also includes most of the reported pathological mutations. Future work on pre-incubated Syn fragments will be necessary to validate this as tubulin-binding domain. Thus, our data provide mechanistic insights into the molecular pathology of PD, which would involve MT-dependent pathways. Indeed, tubulin aggregation induced by mutated Syns (Fig. 6, Supplementary Fig. S2, Supplementary Fig. S8 and Alim *et al*.^9^), could lead to excessive MT bundles, engulfing axons and impairing intracellular transport, as previously described for other neurodegenerative disease^37^. Therefore, axonal transport disruption could be the missing link between Syn/tubulin interaction and PD. In accordance, a very recent paper showed that Syn oligomers, the most toxic Syn species, reduce MT stability, kinesin/MTs interplay and neuritic kinesin-dependent cargoes, promoting early neurite pathology^5^. Although alteration of axonal transport is considered as one of the earliest events in neurodegeneration^38^, we showed that it follows MT dysfunction in the 1-methyl-4-phenyl-1,2,3,6-tetrahydropiridine-induced model of PD^39^,^40^. Hence, by affecting axonal transport, MT dysfunction may trigger the chain of events leading to PD. Accordingly, MT-targeted molecules have beneficial effects in both 1-methyl-4-phenyl-1,2,3,6-tetrahydropiridine-treated^40^ and Syn-overexpressing^41^ mice and the correction of MT defects rescues control phenotype and cell homeostasis in PD patient derived cell lines^42^,^43^,^44^. Therefore, by providing insights into the interaction between Syn and MTs, our data would highlight some essential steps in neuronal function and degeneration.

Many controversial issues remain to be clarified, such as solving the puzzle of the actual naïve state of Syn: is it a folded tetramer^45^ or a labile unstructured monomer^16^? Nevertheless, here we have demonstrated that monomeric WT Syn forms a specific complex with the tubulin α_2_β_2_ tetramer, acquiring a defined secondary structure. We could speculate that local accumulation (i.e. at the pre-synapse) of free tubulin dimers would change the equilibrium between unfolded and folded/tetrameric Syn, which may have evolved to act as a sensor of the tubulin dimers/MTs ratio. Thus, the increase in tubulin dimers concentration and the consequent folding of Syn molecules start the Syn-mediated regulation of both MT nucleation and dynamics, as we have demonstrated here. Therefore, we propose that Syn can be considered a MT dynamase, which potentially set MT mass at the pre-synapse.

## Methods

### Protein purification

Tubulin was purified by two cycles of polymerization/depolymerization in high molar Pipes buffer^13^, suspended in BRB buffer (80 mM K-Pipes, pH 6.9, 2 mM EGTA, 1 mM MgCl_2_) and stored at −80 °C. Recombinant Syn was produced and purified according to Martinez *et al*.^46^, and recombinant RB3-SLD (kindly gifted by prof. Patrick A. Curmi, Evry University, France) was produced and purified according to Charbaut *et al*.^47^ (see also Supplementary information). Aliquots of Syn (in 20 mM Hepes, pH 7.4, 100 mM KCl) and RB3-SLD (in 10 mM Hepes, pH 7.2,150 mM NaCl) were kept at −80 °C until needed. Each aliquot was clarified by ultracentrifugation (230000× *g* at 4 °C for 30 min) immediately before use.

### MT self-assembly

The kinetics of tubulin polymerization was followed turbidimetrically at 350 nm in a multimode plate reader (Infinite 200Pro, Tecan, Mannedorf, Switzerland) equipped with a temperature controller, as previously described^48^. Tubulin was diluted to different concentrations in standard assembly buffer and kept on ice; as WT or mutated Syns were added, the reaction was started by warming the solution at 37 °C. For the preincubation, two solutions were prepared: T1, buffer with 2x glycerol and GTP and w/o proteins, and T2, with double of the final protein concentration but w/o glycerol and GTP. T2 was incubated 10 min at 20 °C, and the reaction was started mixing T1 and T2 1:1 and raising the temperature to 37 °C. Polymerization time-course was dissected and the kinetic parameters calculated according to Bonfils *et al*.^20^: *P* (number of successive steps during nucleation) was determined by plotting log(A(t)/A∞) against log(t) and extrapolated as the pendency of the linear part of the resulting plot; initial velocity of polymerization (V_i_) was calculated as the maximal variation of mass *versus* time (δA/δt); MT assembly was deduced from the extent of absorbance variation (ΔA) at the steady-state. The x-intercept of the linear dependence of ΔA from the initial tubulin concentration represents the tubulin critical concentration. To evaluate MT length and number, 2.7 μM rhodamine-labelled tubulin (Cytoskeleton, Denver, CO) was included in the polymerization solutions and the reaction was stopped by addition of 0.5% glutaraldehyde. MTs were laid on slides and their length was measured using a digital image processing software (Axiovision, Carl Zeiss, Oberkochen, Germany) to analyse the images acquired with the Axiovert 200 M microscope (Carl Zeiss). To assess the ultrastructure of assembled MTs, samples were fixed with 0.5% glutaraldehyde and then placed on Formvar-coated nickel grids, negative stained with uranyl acetate and observed with a Philips CM10 transmission electron microscope at 80 kV, equipped with a Morada Olympus digital camera.

### Co-sedimentation assay

MTs were polymerized (from tubulin 40 μM) 20 min at 37 °C, stabilized 10 min with paclitaxel (Sigma-Aldrich) and then diluted to 4 μM. Syn (0.5–32 μM) was incubated 20 min at 37 °C in the absence or in the presence of MTs and then centrifuged at 70000× *g* for 15 min at 25 °C. Supernatant and pellet were loaded on SDS-PAGE, proteins were transferred onto PVDF membranes and immunostained with anti-Syn rabbit IgG (Sigma-Aldrich) HRP goat anti-rabbit IgG (Sigma-Aldrich). Immunostaining was revealed by enhanced chemiluminescence (Super-Signal West Pico Chemiluminescent, Pierce). Quantification was performed by Image J software (NIH) subtracting the background around bands. According to Ackmann *et al*.^12^, [Syn]_bound_ was plotted *versus* [Syn]_free_ and the data fitted by nonlinear regression to a standard binding equation (Equation 1) using SigmaPlot (Jandel, CA):


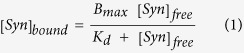


### Mass spectrometry

Nano-ESI-MS was performed on a hybrid quadrupole-time-of-flight instrument (QSTAR Elite, Applied Biosystems, Foster City, CA) with minor modifications to previously reported conditions^49^. Purified proteins were thawed and buffer exchanged by two cycles of desalting on Micro Bio-Spin™ P-6 Gel columns (Bio-Rad laboratories, Hercules, CA), immediately before use. All spectra were acquired in 10 mM ammonium acetate after incubation at room temperature for at least 10 min and no longer than 1 h.

### Circular dichroism

Circular dichroism spectra were acquired using a Jasco J810 spectropolarimeter. Secondary structure of Syn (0.2 mg/ml) and RB3-SLD (0.1 mg/ml), either alone or in the presence of tubulin (1.4 mg/ml), was investigated by recording far-UV circular dichroism spectra in 0.1 cm quartz cuvettes. All proteins were dissolved in BRB buffer. Spectra of pure Syn and RB3-SLD were baseline-corrected by subtracting a buffer spectrum, while difference spectra of the Syn/tubulin and RB3-SLD/tubulin mixtures were “tubulin-corrected” by subtracting the spectrum of the pure tubulin from those of the mixtures. Syn spectra were normalized in terms of mean residual ellipticity by using a mean residue weight of 103 Da. Since BRB buffer does not allow to record spectra below 215 nm, the α-helical content was estimated from the mean residual ellipticity at 222 nm according to Chen and Yang^50^.

### NMR spectroscopy

The NMR spectra were recorded on a Bruker AV600 spectrometer (Bruker Spectrospin AG, Rheinstetten, Germany), operating at 600.10 MHz for the ^1^H nucleus and equipped with a standard triple-resonance probe with z-axis gradients. Temperature control was achieved through the spectrometer BVT3000 temperature control unit, using nitrogen gas (flow 270 l/h) pre-cooled with a Bruker BCU20 refrigeration unit. ^1^H-NMR chemical shifts (δ) were measured in ppm, using as reference external sodium 4,4-dimethyl-2-silapentane-1-sulfonate (DSS) set at 0.00 ppm. DOSY (Diffusion Oriented Spectroscopy) measurements were performed at 25 °C on a freshly prepared 27 μM solution of tubulin, dissolved in 0.6 ml H_2_O:D_2_O 9:1 (v/v), pH 6.8 50 mM phosphate buffer, in the presence of various amounts of Syn (Syn/tubulin molar ratios varied between 0 and 10) after at least 0.5 h incubation. DSS, deuterium oxide (99.8% purity) and 5 mm O.D. NMR tubes (Wilmad 535-PP type) were purchased from Sigma-Aldrich. Solvent suppression was achieved by including in the DOSY pulse-sequence a WATERGATE pulse-scheme^51^. A gradient-based stimulated echo bipolar pulse sequence was utilized^52^, with a 0.3 s diffusion delay (“big delta”) and a 1.5 ms gradient pulse length (“little delta”). 32 one-dimensional spectra were collected with a gradient strength varying between 0.67 and 33.4 Gauss/cm. Values for “little delta” and “big delta” parameters were chosen by taking into account also the expected short transverse relaxation rates due to the formation of high molecular weight aggregates. Other relevant acquisition parameters: time-domain: 2 K; number of scans: 196; relaxation delay: 2 s. Raw data were Fourier-transformed after apodization with a 90°-shifted sine-bell-squared function and baseline corrected. Log(D) values were derived by a two-components non-linear fitting and displayed as pseudo-2D spectra.

Limiting values for the molar fraction of free Syn (α) were estimated from the experimental diffusion coefficients (D, Equation 2) as:


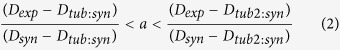


where *D*_*syn*_ is the experimental value determined for free Syn. *D*_*tub:syn*_ and *D*_*tub2:syn*_ are the D values expected for complexes formed by Syn with αβ tubulin dimer and α_2_β_2_ tubulin tetramer, respectively. These last values were estimated by applying the following correction factors to *D*_*tub*_:


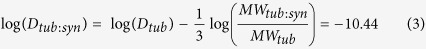



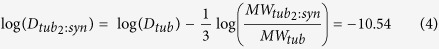


The dissociation constant K_d_ of Syn-tubulin complex was derived by applying the following equation 5:


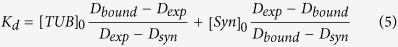


where *D*_*bound*_ can be either *D*_*tub:syn*_ or *D*_*tub2:syn*_ as derived from equation 3 and 4.

### Video-microscopy and data analysis

MTs were assembled from purified axonemes with tubulin (10–15 μM) and increasing concentrations of preincubated Syn (5–15 μM). Samples were prepared in perfusion chambers, previously saturated with 50 μM Syn, and observed at 37 °C with an Olympus BX-51 microscope equipped with DIC prisms and a video camera coupled to an Argus 20 image processor (Hamamatsu, Hamamatsu City, Japan), as previously described^53^. Images were recorded every 2 s over periods of 5 min. The total recording time did not exceed 60 min for each chamber. Measurements of MT dynamics and data analysis were carried out using Image J (NIH, Bethesda, MD) and Kaleidagraph (Synergy Software Systems, Dubai, UAE), as previously described^53^.

### Immunoblotting and live cell imaging

PC12 cells were maintained in cultures and differentiated as previously described^39^. PC12 cells were transiently transfected using Lipofectamine 2000 (Invitrogen), with C-terminal GFP-fused human WT Syn or with GFP-containing control vector (eGFP-N1). Whole cell extracts were loaded on SDS-PAGE, transferred onto PVDF membranes and immunostained with anti-Syn rabbit IgG and anti-GAPDH as loading control. For live cell imaging experiments, GFP-WT Syn and GFP-containing vectors were co-transfected with EB3-mCherry construct^24^ (kindly provided by Dr Galjart, Medical Genetic Center, Erasmus University, Rotterdam, The Netherlands). As previously described^39^, cultures were transferred to a live cell imaging workstation; images were collected every 6 s with a cooled camera (Axiocam HRM Rev. 2; Zeiss) for periods of 3-4 min, and the total recording time did not exceed 60 min for each dish. MT growth dynamics was automatically analysed using plusTipTracker software^54^. The area of the analysed neurites was estimated by ImageJ software (Supplementary Fig. S6).

### Protein alignment

Pairwise alignments between Syn and stathmin sequences were made using Pam250 or Bl50 matrix, whereas multiple protein alignment was performed with ClustalW software.

### Statistical analyses

For multiple comparisons, the statistical significance of treatment was assessed by one-way ANOVA with Dunnett 2-sided or Fischer LSD *post-hoc* testing, whereas differences between WT Syn and controls were assessed using Student’s t-test. All analyses were performed using STATISTICA (StatSoft Inc., Tulsa, OK).

## Additional Information

**How to cite this article**: Cartelli, D. *et al*. α-Synuclein is a Novel Microtubule Dynamase. *Sci. Rep.* **6**, 33289; doi: 10.1038/srep33289 (2016).

## Supplementary Material

Supplementary Information

## Figures and Tables

**Table 1:** Folded Syn increases MT dynamics *in vitro*.

	Tub 10 μM			Tub 15 μM			
	Syn 0 μM	Syn 5 μM	Syn 10 μM	Syn 0 μM	Syn 5 μM	Syn 10 μM	Syn 15 μM
V_growth_ (μm/min)	1.2 ± 0.1	1.38 ± 0.5	1.08 ± 0.18	1.1 ± 0.086	1.17 ± 0.14	1.38 ± 0.14*	1.32 ± 0.24*
	n = 1097	n = 362	n = 395	n = 1420	n = 663	n = 695	n = 973
V_shrinkage_ (μm/min)	8.7 ± 2.24	7.35 ± 2.1	7.5 ± 1.65	11 ± 1.77	8.6 ± 2	11.7 ± 2.6	8.5 ± 2.24
	n = 357	n = 209	n = 309	n = 287	n = 216	n = 298	n = 336
F_catastrophe_ (min^−1^)	0.16 ± 0.05	1.04 ± 0.3	0.69 ± 0.17	0.09 ± 0.03	0.27 ± 0.07	0.26 ± 0.07	0.22 ± 0.06
	n = 9	n = 12	n = 15	n = 9	n = 14	n = 12	n = 11
F_rescue_ (min^−1^)	0.15	0.67 ± 0.33	0.33 ± 0.19	0.1	0.6 ± 0.26	0.44 ± 0.22	1.26 ± 0.44
	n = 1	n = 4	n = 3	n = 1	n = 5	n = 4	n = 8
Growth Duration (s)	3327	692	1304	5797	3056	2751	2944
Shrinkage Duration (s)	382	356	537	594	512	540	380
MT number	32	14	16	41	22	25	20

Dynamic parameters were determined by VE-DIC light microscopy for MTs assembled from purified axonemes in the presence of tubulin (10 and 15 μM) and increasing concentrations of Syn (0–15 μM). In each condition, Syn was preincubated with tubulin prior the observation at 37 °C. Velocities are expressed as mean ± SEM. The total growth and shrinkage times analysed, as well as the number of MTs used for each condition, are given in the last three rows. Catastrophe and rescue frequencies were calculated by dividing the total number of events by the time spent in growth and shrinkage, respectively. The standard deviation is calculated by dividing F_catastrophe_ or F_rescue_ by √n assuming a Poisson distribution^55^. (*n*) represents the total number of measurements for the growth and shrinkage rates, and the total number of observed events for the catastrophe and rescue frequencies. *p < 0.05 vs Syn 0 μM, according to ANOVA, Dunnett *post hoc* test. Actual p are 0.005 (Syn 10 μM) and 0.0051 (Syn 15 μM).

## References

[b1] Conde C. & Cáceres A. Microtubule assembly, organization and dynamics in axons and dendrites. Nat. Rev. Neurosci. 10, 319–332 (2009). 19377501 10.1038/nrn2631

[b2] Stiess M. . Axon extension occurs independently of centrosomal microtubule nucleation. Science 327, 704–707 (2010). 20056854 10.1126/science.1182179

[b3] Lashuel H. A., Overk C. R., Oueslati A. & Masliah E. The many faces of α-synuclein: from structure and toxicity to therapeutic target. Nat. Rev. Neurosci. 14, 38–48 (2013). 23254192 10.1038/nrn3406 PMC4295774

[b4] Winner B. . *In vivo* demonstration that alpha-synuclein oligomers are toxic. Proc Natl Acad Sci USA 108, 4194–4199 (2001). 21325059 10.1073/pnas.1100976108 PMC3053976

[b5] Prots I. . α-Synuclein oligomers impair neuronal microtubule-kinesin interplay. J. Biol. Chem. 288, 21742–21754 (2013). 23744071 10.1074/jbc.M113.451815 PMC3724632

[b6] Alim M. A. . Tubulin seeds alpha-synuclein fibril formation. J. Biol. Chem. 277, 2112–2117 (2002). 11698390 10.1074/jbc.M102981200

[b7] Esteves A. R., Arduíno D. M., Swerdlow R. H., Oliveira C. R. & Cardoso S. M. Microtubule depolymerization potentiates alpha-synuclein oligomerization. Front. Aging Neurosci. 1, 5 (2010). 20552056 10.3389/neuro.24.005.2009 PMC2874407

[b8] Nakayama K., Suzuki Y. & Yazawa I. Microtubule depolymerization suppresses alpha-synuclein accumulation in a mouse model of multiple system atrophy. Am. J. Pathol. 174, 1471–1480 (2009). 19286568 10.2353/ajpath.2009.080503 PMC2671377

[b9] Alim M. A. . Demonstration of a role for alpha-synuclein as a functional microtubule-associated protein. J. Alzheimers Dis. 6, 435–442 discussion 443–449 (2004). 15345814 10.3233/jad-2004-6412

[b10] Chen L. . Oligomeric alpha-synuclein inhibits tubulin polymerization. Biochem. Biophys. Res. Commun. 356, 548–553 (2007). 17374364 10.1016/j.bbrc.2007.02.163

[b11] Payton J. E., Perrin R. J., Clayton D. F. & George J. M. Protein-protein interactions of alpha-synuclein in brain homogenates and transfected cells. Brain Res. Mol. Brain Res. 95, 138–145 (2001). 11687285 10.1016/s0169-328x(01)00257-1

[b12] Ackmann M., Wiech H. & Mandelkow E. Nonsaturable Binding Indicates Clustering of Tau on the Microtubule Surface in a Paired Helical Filament-like Conformation. J. Biol. Chem. 275, 30335–30343 (2000). 10869348 10.1074/jbc.M002590200

[b13] Castoldi M. & Popov A. V. Purification of brain tubulin through two cycles of polymerization-depolymerization in a high-molarity buffer. Protein Expr. Purif. 32, 83–88 (2003). 14680943 10.1016/S1046-5928(03)00218-3

[b14] Testa L. . Electrospray ionization-mass spectrometry conformational analysis of isolated domains of an intrinsically disordered protein. Biotechnol. J. 6, 96–100 (2011). 21053335 10.1002/biot.201000253

[b15] Weinreb P. H., Zhen W., Poon A. W., Conway K. A. & Lansbury P. T. Jr. NACP, a protein implicated in Alzheimer’s disease and learning, is natively unfolded. Biochemistry 35, 13709–13715 (1996). 8901511 10.1021/bi961799n

[b16] Burré J. . Properties of native brain α-synuclein. Nature 498, E4–6 discussion E6-7 (2013). 23765500 10.1038/nature12125 PMC4255827

[b17] Eliezer D., Kutluay E., Bussell R. Jr & Browne G. Conformational properties of alpha-synuclein in its free and lipid-associated states. J. Mol. Biol. 307, 1061–1073 (2001). 11286556 10.1006/jmbi.2001.4538

[b18] Steinmetz M. O. Structure and thermodynamics of the tubulin-stathmin interaction. J. Struct. Biol. 158, 137–147 (2007). 17029844 10.1016/j.jsb.2006.07.018

[b19] Uversky V. N., Li J. & Fink A. L. Evidence for Partially Folded Intermediate in α-Synuclein Fibril Formation. J. Biol. Chem. 276, 10737–10744 (2001). 11152691 10.1074/jbc.M010907200

[b20] Bonfils C., Bec N., Lacroix B., Harricane M. C. & Larroque C. Kinetic analysis of tubulin assembly in the presence of the microtubule-associated protein TOGp. J. Biol. Chem. 282, 5570–5581 (2007). 17178729 10.1074/jbc.M605641200 PMC2238798

[b21] Hiller G. & Weber K. Radioimmunoassay for tubulin: a quantitative comparison of the tubulin content of different established tissue culture cells and tissues. Cell 14, 795–804 (1978). 688394 10.1016/0092-8674(78)90335-5

[b22] Kamp F. . Inhibition of mitochondrial fusion by α-synuclein is rescued by PINK1, Parkin and DJ-1. EMBO J. 29, 3571–3589 (2010). 20842103 10.1038/emboj.2010.223 PMC2964170

[b23] Stefanis L., Kholodilov N., Rideout H. J., Burke R. E. & Greene L. A. Synuclein-1 is selectively up-regulated in response to nerve growth factor treatment in PC12 cells. J. Neurochem. 76, 1165–1176 (2001). 11181836 10.1046/j.1471-4159.2001.00114.x

[b24] Komarova Y. . Mammalian end binding proteins control persistent microtubule growth. J. Cell Biol. 184, 691–706 (2009). 19255245 10.1083/jcb.200807179 PMC2686402

[b25] Uversky V. N. Neuropathology, biochemistry, and biophysics of alpha-synuclein aggregation. J. Neurochem. 103, 17–37 (2007). 17623039 10.1111/j.1471-4159.2007.04764.x

[b26] Zhou R. M. . Molecular interaction of α-synuclein with tubulin influences on the polymerization of microtubule *in vitro* and structure of microtubule in cells. Mol. Biol. Rep. 37, 3183–3192 (2010). 19826908 10.1007/s11033-009-9899-2

[b27] Kara E. . α-Synuclein mutations cluster around a putative protein loop. Neurosci. Lett. 546, 67–70 (2013). 23669636 10.1016/j.neulet.2013.04.058 PMC3694303

[b28] Honnappa S., Cutting B., Janke W., Seelig J. & Steinmetz M. O. Thermodynamics of Op18/stathmin-tubulin interaction. J. Biol. Chem. 278, 38926–38934 (2003). 12860982 10.1074/jbc.M305546200

[b29] Feng Y. & Walsh C. A. Protein-protein interactions, cytoskeletal regulation and neuronal migration. Nat. Rev. Neurosci. 2, 408–416 (2001). 11389474 10.1038/35077559

[b30] Erent M., Drummond D. R. & Cross R. A. S. pombe kinesins-8 promote both nucleation and catastrophe of microtubules. PLoS One. 7, e30738 (2012). 22363481 10.1371/journal.pone.0030738 PMC3282699

[b31] Gardner M. K., Zanic M., Gell C., Bormuth V. & Howard J. Depolymerizing kinesins Kip3 and MCAK shape cellular microtubule architecture by differential control of catastrophe. Cell 147, 1092–1103 (2011). 22118464 10.1016/j.cell.2011.10.037

[b32] Mozziconacci J., Sandblad L., Wachsmuth M., Brunner D. & Karsenti E. Tubulin dimers oligomerize before their incorporation into microtubules. PLoS One 3, e3821 (2008). 19043587 10.1371/journal.pone.0003821 PMC2584370

[b33] Schek H. T. 3rd, Gardner M. K., Cheng J., Odde D. J. & Hunt A. J. Microtubule assembly dynamics at the nanoscale. Curr. Biol. 17, 1445–1455 (2007). 17683936 10.1016/j.cub.2007.07.011 PMC2094715

[b34] Slep K. C. & Vale R. D. Structural basis of microtubule plus end tracking by XMAP215, CLIP-170, and EB1. Mol. Cell. 27, 976–991 (2007). 17889670 10.1016/j.molcel.2007.07.023 PMC2052927

[b35] Manna T., Thrower D. A., Honnappa S., Steinmetz M. O. & Wilson L. Regulation of microtubule dynamic instability *in vitro* by differentially phosphorylated stathmin. J. Biol. Chem. 284, 15640–15649 (2009). 19359244 10.1074/jbc.M900343200 PMC2708860

[b36] Gasser T., Hardy J. & Mizuno Y. Milestones in PD genetics. Mov. Disord. 26, 1042–1048 (2011). 21626549 10.1002/mds.23637

[b37] Salinas S., Carazo-Salas R. E., Proukakis C., Schiavo G. & Warner T. T. Spastin and microtubules: Functions in health and disease. J. Neurosci. Res. 85, 2778–2782 (2007). 17348041 10.1002/jnr.21238

[b38] Morfini G. A. . Axonal transport defects in neurodegenerative diseases. J. Neurosci. 29, 12776–12786 (2009). 19828789 10.1523/JNEUROSCI.3463-09.2009 PMC2801051

[b39] Cartelli D. . Microtubule dysfunction precedes transport impairment and mitochondria damage in MPP+-induced neurodegeneration. J. Neurochem. 115, 247–258 (2010). 20649848 10.1111/j.1471-4159.2010.06924.x

[b40] Cartelli D. . Microtubule alterations occur early in experimental parkinsonism and the microtubule stabilizer epothilone D is neuroprotective. Sci. Rep. 3, 1837 (2013). 23670541 10.1038/srep01837 PMC3653217

[b41] Fleming S. M. . A pilot trial of the microtubule-interacting peptide (NAP) in mice overexpressing alpha-synuclein shows improvement in motor function and reduction of alpha-synuclein inclusions. Mol. Cell Neurosci. 46, 597–606 (2011). 21193046 10.1016/j.mcn.2010.12.011 PMC3046337

[b42] Cartelli D., Goldwurm S., Casagrande F., Pezzoli G. & Cappelletti G. Microtubule destabilization is shared by genetic and idiopathic Parkinson’s disease patient fibroblasts. PLoS One 7, e37467 (2012). 22666358 10.1371/journal.pone.0037467 PMC3359730

[b43] Esteves A. R., Gozes I. & Cardoso S. M. The rescue of microtubule-dependent traffic recovers mitochondrial function in Parkinson’s disease. Biochim. Biophys. Acta. 1842, 7–21 (2014). 24120997 10.1016/j.bbadis.2013.10.003

[b44] Ren Y. . Parkin Mutations Reduce the Complexity of Neuronal Processes in iPSC-derived Human Neurons. Stem Cells 33, 68–78 (2015). 25332110 10.1002/stem.1854 PMC4429885

[b45] Bartels T., Choi J. G. & Selkoe D. J. α-Synuclein occurs physiologically as a helically folded tetramer that resists aggregation. Nature 477, 107–110 (2011). 21841800 10.1038/nature10324 PMC3166366

[b46] Martinez J., Moeller I., Erdjument-Bromage H., Tempst P. & Lauring B. Parkinson’s disease-associated alpha-synuclein is a calmodulin substrate. J. Biol. Chem. 278, 17379–17387 (2003). 12610000 10.1074/jbc.M209020200

[b47] Charbaut E. . Stathmin family proteins display specific molecular and tubulin binding properties. J. Biol. Chem. 276, 16146–16154 (2001). 11278715 10.1074/jbc.M010637200

[b48] Contini A., Cappelletti G., Cartelli D., Fontana G. & Gelmi M. L. Molecular dynamics and tubulin polymerization kinetics study on 1,14-heterofused taxanes: evidences of stabilization of the tubulin head-to-tail dimer-dimer interaction. Mol. Biosyst. 8, 3254–3261 (2012). 23073462 10.1039/c2mb25326g

[b49] Santambrogio C. . DE-loop mutations affect beta2 microglobulin stability, oligomerization, and the low-pH unfolded form. Protein Sci. 19, 1386–1397 (2010). 20506535 10.1002/pro.419 PMC2974830

[b50] Chen Y. H. & Yang J. T. A new approach of the calculation of secondary structures of globular proteins by optical rotatory disoersion and circular dichroism. Biochem. Biophys. Res. Commun. 44, 1285–1291 (1971). 5168596 10.1016/s0006-291x(71)80225-5

[b51] Piotto M., Saudek V. & Sklenar V. Gradient-tailored excitatiojn for single-quantum NMR spectroscopy of aqueous solutions. J. Biomol. NMR 2, 661–665 (1992). 1490109 10.1007/BF02192855

[b52] Wu D., Chen A. & Johnson C. S. Jr. An Improved Diffusion-Ordered Spectroscopy Experiment Incorporating Bipolar-Gradient Pulses. J. Magn. Reson. Series A 108, 255–258 (1995).

[b53] Vitre B. . EB1 regulates microtubule dynamics and tubulin sheet closure *in vitro*. Nat. Cell Biol. 10, 415–421 (2008). 18364701 10.1038/ncb1703

[b54] Applegate K. T. . plusTipTracker: Quantitative image analysis software for the measurement of microtubule dynamics. J. Struct. Biol. 176, 168–184 (2011). 21821130 10.1016/j.jsb.2011.07.009 PMC3298692

[b55] Walker R. A. . Dynamic instability of individual microtubules analysed by video light microscopy: rate constants and transition frequencies. J. Cell Biol. 107, 1437–1448 (1988). 3170635 10.1083/jcb.107.4.1437 PMC2115242

