# Supplementary material for: α-Synuclein is a Novel Microtubule Dynamase
Source: Sci Rep. 2016 Sep 15;6:33289. doi: 10.1038/srep33289 (PMC5024109; doi:10.1038/srep33289)

# α-SYNUCLEIN IS A NOVEL MICROTUBULE DYNAMASE

Daniele Cartelli[1,§], Alessandro Aliverti[1], Alberto Barbiroli[2], Carlo Santambrogio[3], Enzio M. Ragg[2], Francesca V.M. Casagrande[1], Francesca Cantele[1], Silvia Beltramone[1], Jacopo Marangon[1], Carmelita De Gregorio[1], Vittorio Pandini[1], Marco Emanuele[4], Evelina Chieregatti[4], Stefano Pieraccini[5], Staffan Holmqvist[6,7], Luigi Bubacco[8], Laurent Roybon[6,7], Gianni Pezzoli[9], Rita Grandori[3], Isabelle Arnal[10], Graziella Cappelletti[1,11*]

[1]Dept. Biosciences, Università degli Studi di Milano, Milano (Italy);
[2]Dept. of Food, Environmental and Nutritional Sciences, Università degli Studi di Milano, Milano (Italy);
[3]Dept. Biotecnology and Biosciences, Università degli Studi di Milano-Bicocca, Milano (Italy);
[4]Dept. Neuroscience and Brain Technologies, Istituto Italiano di Tecnologia, Genova (Italy);
[5]Dept. Chemistry, Università degli Studi di Milano, Milano (Italy);
[6] Stem Cell laboratory for CNS Disease Modeling, Wallenberg Neuroscience Center, Department of Experimental Medical Science, Lund University, Lund, Sweden;
[7] Strategic Research Area MultiPark and Lund Stem Cell Center, Lund University, Lund, Sweden
[8]Dept. Biology, University of Padova, Padova (Italy)
[9]Parkinson Institute, Istituti Clinici di Perfezionamento, Milano (Italy);
[10]Grenoble Institut des Neurosciences, Grenoble (France);
[11]Center of Excellence on Neurodegenerative Diseases, Università degli Studi di Milano, Milano (Italy).

**SUPPLEMENTARY METHODS**

**Protein purification**

Recombinant Syn or RB3-SLD was overproduced in *Escherichia coli* using the plasmid constructs and culturing conditions reported by Martinez et al.[46] or by Charbaut et al.[47], respectively. For the isolation of both proteins, bacterial pellets were lysed by sonication and followed by incubation at 90 °C for 5 min. After removal of cell debris and denatured proteins by centrifugation, supernatants were subjected to ion exchange chromatography on a Q Sepharose HP column (GE Healthcare, Uppsala, Sweden). Aliquots of Syn (in 20 mM Hepes, pH 7.4, 100 mM KCl) and RB3-SLD (in 10 mM Hepes, pH 7.2,150 mM NaCl) were snap-frozen in liquid nitrogen and kept at -80 °C until needed.

**Differential interference contrast microscopy**

To verify the capability of Syn to co-polymerize with MTs, the sedimentable fraction obtained by centrifugation of tubulin following assembly in the presence of Syn was gently resuspended in BRB buffer, laid on poly-L-lysine coated coverslips, and immunostained with anti-Syn rabbit IgG (Sigma-Aldrich, St. Louis, MO) and Alexa Fluor™ 488-labeled goat anti-rabbit IgG (Invitrogen, Carlsbad, CA,) antibodies. The coverslips were mounted in Mowiol® (Calbiochem, San Diego, CA)–DABCO (Sigma-Aldrich) and examined with an Axiovert 200M microscope (Carl Zeiss, Oberkochen, Germany), using differential interference contrast (DIC) optics to observe MTs and fluorescence to visualize Syn staining.

**Western blotting on pure proteins**

Purified tubulin (30 μg) from two different batches and WT Syn (10 or 100 ng) were loaded on 12% SDS-PAGE; proteins were transferred onto PVDF membranes and immunostained with anti-Syn rabbit IgG (Sigma-Aldrich), monoclonal anti-Syn mouse IgG (clone 4D6, Abcam) or anti-α tubulin mouse IgG (clone B-5-1-2, Sigma-Aldrich) Alexa Fluor™ 488 donkey anti-rabbit or Alexa Fluor™ 568 goat anti-mouse IgG (Invitrogen). Acquisitions were performed by ChemiDoc and Image Lab software (Bio-Rad, Hercules, CA).

**Differentiation of human embryonic stem cells into midbrain neurons**

The differentiation of the human embryonic stem cells (line HuES13= H13) toward mesencephalic neurons was performed according to previous published protocol[56] with the following modifications: LDN-193189 (100 nM, Stemgent) was employed instead of Noggin, and CHIR was kept in differentiation medium together with neurotrophic factors until embryoid bodies were dissociated and seeded onto coated surfaces, at 30DIV. Three days later cultures were fixed and processed for immunocytochemistry.

**Immunofluorescence**

PC12 cells or neurons were fixed in cold methanol (6 min at -20 °C), saturated 15 min with 5% BSA and stained with anti-tyrosinated tubulin mouse IgG (clone TUB-1A2, Sigma-Aldrich) or anti-βIII tubulin mouse

IgG (clone SDl.3D10, Sigma-Aldrich) and anti-Syn rabbit IgG for 1h at 37 °C. After washing in PBS, samples were stained with Alexa Fluor™ 568 goat anti-mouse and Alexa Fluor™ 488 donkey anti-rabbit (Invitrogen). Coverslips were mounted in Mowiol®-DABCO and examined with a confocal laser scan microscope imaging system (TCS SP5 AOBS, Leica Microsystems, Heidelberg, Germany) equipped with Ar/Ar-Kr 488 nm, 561 nm and 405 nm diode lasers. Photomultiplier gain for each channel was adjusted to minimize background noise and saturated pixels and parameters were kept constant for all the acquisitions. To estimate the co-localization area between red and green signals, analyses were carried out on single-plane raw images and Manders' coefficients were calculated using the JACoP plug-in for Image J software[57] and the random overlap has been controlled for by manually and randomly translating pixels (less than 5% in term of image dimension, by using the *ad hoc* ImageJ operation) either in the green image (Syn) or in the red one (Tubulins). To further strengthen our results, we performed the analyses of co-localization by using the r parameter (Pearson coefficient) on the original images or by applying the Costes' randomization. To do that, we used the appropriate module of the JACOP plugin and the following settings (automatically chosen by the software): number of randomization rounds = 1000; resolution, bin width = 0.001. All the values for the co-localization parameters are reported as mean ± SEM.

**Size exclusion chromatography – Multi Angle Light Scattering**

Purified Synuclein, WT and mutants, were characterized by a Size Exclusion-HPLC system equipped with a Waters 2487 Dual λ Absorbance Detector and a Optilab T-rEX Refractive Index Detector (Wyatt, Santa Barbara, CA, USA), connected in-line with a Dawn Heleos Multi Angle Light Scattering (Wyatt). 200 µl of 0.2 g/l samples were separated in a Superose 12 10/300 GL column (mobile phase 20 mM Hepes, 100 mM KCl, pH 7.4; flow 0.5 ml/min) and molar mass of eluted peaks was calculated by means of Astra software (v. 5.3.4.18, Wyatt) by using 0.185 as dn/dc value.

56. Kirkeby, A., Nelander, J. & Palmar, M. Generating regionalized neuronal cells from pluripotency, a step-by-step protocol. *Front. Cell Neurosci*. **6**, 64 (2013).
57. Bolte, S. & Cordelières, F.P. A guided tour into subcellular co-localization analysis in light microscopy. *J. Microsc*. **224**, 213-232 (2006).

**Supplementary Figure S1. Tubulin quantification.** Histogram showing the amount of tubulin recovered in the pellet (P, white bars) or supernatant (S, black bars) fractions of the co-sedimentation experiments performed with different concentrations of WT Syn (0-32 µM). The data reported derive from at least three different replicates, and are expressed as mean ± SEM. The analyses refer to data shown in Fig. 1a.

**Supplementary Figure S2. Syn interacts with MTs.** (a) MTs assembled *in vitro* (from 40 µM of tubulin) in the presence of 5 µM of WT (WT) or 5 µM of mutated (A30P and A53T) Syn are observed by DIC microscopy, and stained with anti-Syn (Syn) antibody (green). Scale bar, 2 µm. (b) Western blotting showing the presence of Syn in two different batches of purified tubulin (Tub A and Tub B, 30 µg per lane). Recombinant WT Syn was used as positive control (Syn) in blot immunostained with polyclonal (loaded 10 ng) and monoclonal (loaded 100 ng) anti-Syn antibodies and recombinant neuroserpin as negative control (NSP).

**Supplementary Figure S3. Rhodamine-labelled MTs.** (a) Tubulin assembly was recorded over time by measuring the increase in absorbance variation (ΔA) at 350 nm. Tubulin (40 µM) was polymerized in the absence (Tub alone, solid grey line) and in the presence of 40 µM of preincubated WT Syn (Tub + Syn, solid black line). To exclude the formation of Syn aggregates, 40 µM of preincubated WT Syn (Syn alone, dashed black line) was also monitored over the same time. (b) Electron microscope images of MTs collected at the end of assembly kinetics shown in (a) and assembled in the absence (Tub alone) or in the presence of Syn (Tub + Syn), and of Syn incubated in the absence of tubulin (Syn alone). Scale bar, 50 nm. (c) Images of MTs assembled from 40 µM naïve tubulin and 2.7 µM rhodamine-labelled tubulin were captured after 2 and 45 min of polymerization by fluorescent microscope and showed in inverted contrast. The images obtained in the absence (CONT) or in the presence of 5 µM of WT Syn (WT) were analysed to measure the MT length and number (as reported in Figure 3). Scale bar, 2 µm.

**Supplementary Figure S4. WT Syn co-localizes with MTs in murine neurons**. (a) Confocal micrographs of PC12 cells (PC12) differentiated 5 days with NGF expressing human WT GFP-Syn chimeras (green) and stained for tyrosinated tubulin (Tyr TUB, red) or βIII tubulin (βIII TUB, red). The co-localizing pixels are shown (Coloc) as the co-localization index (M= Manders' coefficient of Syn overlapping tubulin). Scale bar, 5 µm. (b) Manders' coefficients of Syn overlapping tubulin (Syn vs TUB, M) obtained on scrambled images (pixels randomly shifted less than 5% in term of the image dimension) and performed translating Syn (M green shifted) or tubulin (M red shifted) signal were compared to M values obtained on the original images. *$p<0.05$ vs (Syn vs TUB) according to Student's t-test. Actual p are: Tyr TUB, green shifted=0.00044 and red shifted= 0.0001; βIII TUB, green shifted= $3.1E^{-07}$ and red shifted= $3.98E^{-07}$. (c) Analyses of co-localization parameter r, obtained on the original images (Pearson original) or applying the Costes' randomization (Costes' randomization). *$p<0.05$ vs Pearson original according to Student's t-test. Actual p are: Tyr TUB= 0.016; βIII TUB= 0.03.

**Supplementary Figure S5. Syn interacts with MTs in human mesencephalic neurons.** (a) Human embryonic stem cell-derived mesencephalic neurons co-express OTX2 and TH. (b) Confocal micrographs of human mesencephalic neurons stained for endogenous Syn (Syn, green) and tyrosinated tubulin (Tyr TUB, red), or βIII tubulin (βIII TUB, red). The co-localizing pixels are shown (Coloc) as the co-localization index (M= Manders' coefficient of Syn overlapping tubulin). Scale bar, 5 μm. (c) Manders' coefficients of Syn overlapping tubulin (Syn vs TUB, M) on scrambled images (pixels randomly shifted less than 5% in term of the image dimension) and performed translating Syn (M green shifted) or tubulin (M red shifted) signal were compared to M values obtained on the original images. *$p<0.05$ vs (Syn vs TUB) according to Student's t-test. Actual p are: Tyr TUB, green shifted=0.006 and red shifted= 0.006; βIII TUB, green shifted= 0.002 and red shifted= 0.003. (d) Analyses of co-localization parameter r, obtained on the original images (Pearson original) or applying the Costes' randomization (Costes' randomization). *$p<0.05$ vs Pearson original according to Student's t-test. Actual p are: Tyr TUB= 0.0003; βIII TUB= 0.0012.

**Supplementary Figure S6. Measurements of neurite areas.** Histogram representing the area of the neurite of PC12 cells expressing GFP (GFP) or GFP-Syn (Syn), differentiated 5 days with NGF, and used to evaluate MT growth by the analyses of EB3 movies in basal conditions (BASAL, cell maintained at 37 °C) and during the rewarming phase (RECOVERY) after 30 min at 4 °C. The analyses refer to the experiments reported in Fig. 4.

**Supplementary Figure S7. Alignment of Syn fragment 1-100.** Multiple alignment of the fragment 1-100 of Syn (Syn, red) with four members of stathmin family was performed by anchoring the fragment centered around the residue 30 or 53 as shown in Fig. 5 (not shown here). The others Syn fragments were aligned to the regions of the stathmin family proteins as follows: Syn 1-19 to the N-terminus of the family (Region 1); Syn 41-42 to the in between Domain 1 and 2 (Region 2); Syn 63-100 to the C-terminus of the family (Region 3). Asterisks mark invariant positions, while dots and colons highlight semi-conservative and conservative substitutions, respectively. SCG= SCG10, SCL= SCLIP, STA= stathmin, RB3=RB3.

**Supplementary Figure S8. Mutated Syns promote protein aggregation.** (a) SEC-MALS chromatograms of WT (WT) and mutated (A53T, E46K, and A30P) Syns. The peak of the chromatograms (elution volume 12 ml) corresponds to a molar mass of 14 kDa. (b) Representative electron micrographs of WT (WT) or mutated (A30P, A53T and E46K) Syns, at the concentration of 5 μM, preincubated at 20°C for 10 min and then incubated at 37°C for 45 in the absence (w/o Tub) or in the presence (+ Tub) of tubulin (40 μM). Scale bar, 100 nm.

**Supplementary Figure S1**

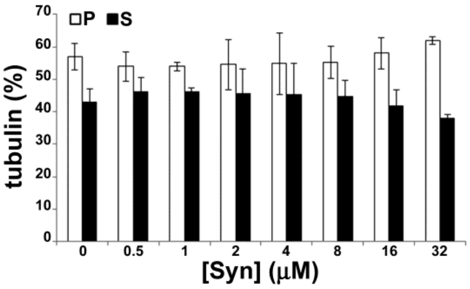

**Supplementary Figure S2**

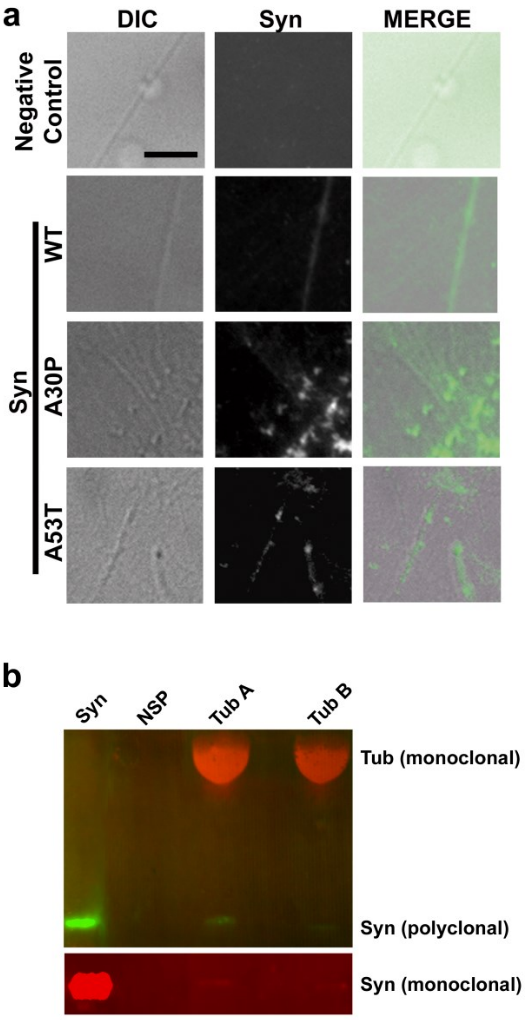

**Supplementary Figure S3**

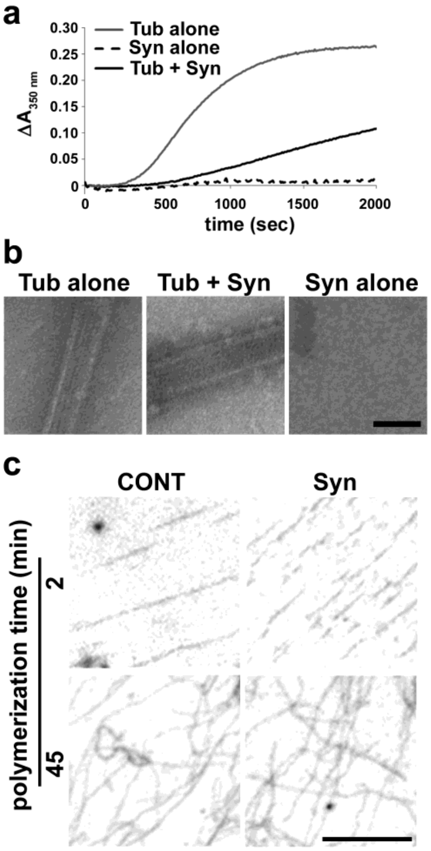

## Supplementary Figure S4

a

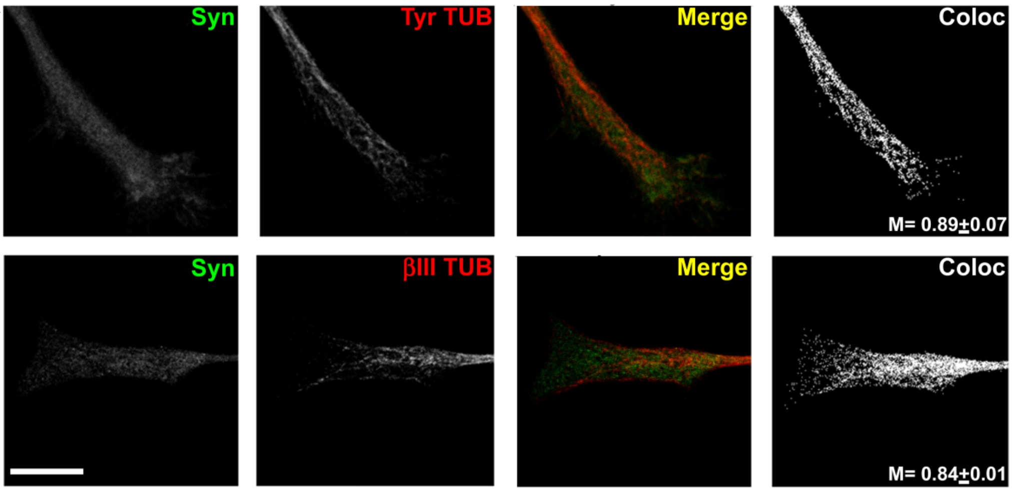


b

| | Syn vs TUB (M) | M green shifted | M red shifted |
|---|---|---|---|
| Tyr Tub | 0.89 ± 0.07 | 0.38 ± 0.06 * | 0.26 ± 0.06 * |
| βIII Tub | 0.84 ± 0.01 | 0.29 ± 0.03 * | 0.22 ± 0.03 * |

c

| | Pearson (original) | Costes' randomization |
|---|---|---|
| Tyr Tub | 0.32 ± 0.08 | 0.0 ± 0.001* |
| βIII Tub | 0.35 ± 0.05 | 0.0 ± 0.0008* |

**Supplementary Figure S5**

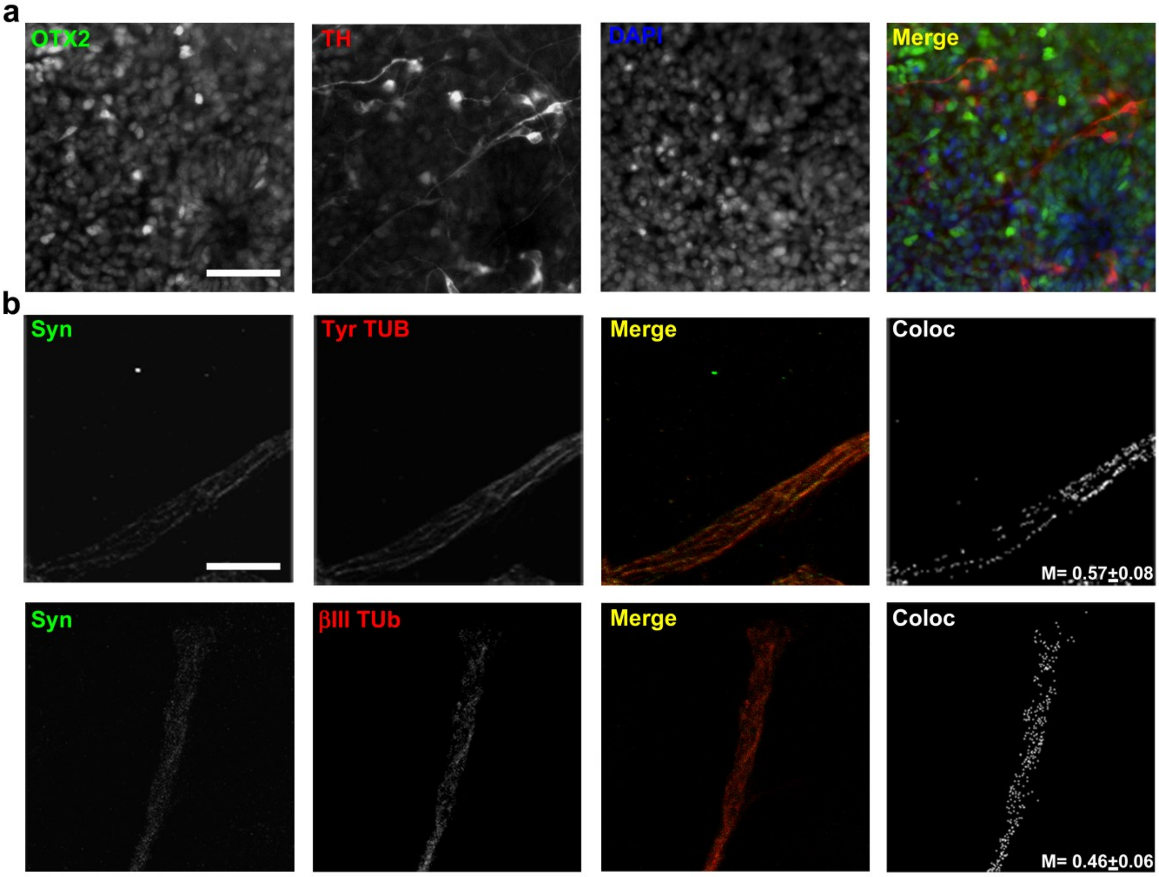


**c**

| | Syn vs TUB (M) | M green shifted | M red shifted |
|---|---|---|---|
| Tyr Tub | 0.57 ± 0.08 | 0.14 ± 0.04 * | 0.15 ± 0.04 * |
| βIII Tub | 0.46 ± 0.06 | 0.18 ± 0.04 * | 0.15 ± 0.04 * |

**d**

| | Pearson (original) | Costes' randomization |
|---|---|---|
| Tyr Tub | 0.37 ± 0.03 | 0.0 ± 0.00014 * |
| βIII Tub | 0.35 ± 0.05 | 0.0 ± 0.0007 * |

**Supplementary Figure S6**

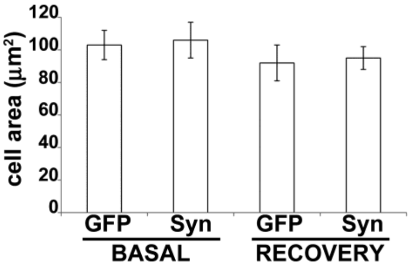

## Supplementary Figure S7

### Region1 (1-19)

```
SCG> MAKTAMAYKEKMKELSMLSLICSCFYPEPRNINIYTYD------------DME
SCL> MASTISAYKEKMKELSVLSLICSCFYTQPHPNTVYQYG------------DME
STA> MASS----------------------------------------------DIQ
RB3> --MTLAAYKEKMKELPLVSLFCSCFLSDPLNKSSYKYEADTVDLNWCVISDME
SYN> -MDVFMKGLSKAKEGVVAA-------------------------------AEK
                                                         :
```

### Region2 (41-42)

```
SCG> PSPIS-EAPRTLASPKKKDLSLEEIQKKLEAAEERRKSQEAQVLKQLAEKREHEREVLQK
SCL> PSDLSPESPMLSSPPKKKDTSLEELQKRLEAAEERRKTQEAQVLKQLAERREHEREVLHK
STA> PSFDG-VPEFNASLPRRRDPSLEEIQKKLEAAEERRKYQEAELLKHLAEKREHEREVIQK
RB3> RSKES-VPEFPLSPPKKKDLSLEEIQKKLEAAEERRKSHEAEVLKQLAEKREHEKEVLQK
SYN> GS----------------------------------------------------------
      *
SCG> ALEENNNFSKMAEEKLILKME
SCL> ALEENNNFSRQAEEKLNYKME
STA> AIEENNNFIKMAKEKLAQKME
RB3> AIEENNNFSKMAEEKLTHKME
SYN> ---------------------
```

### Region3 (64-100)

```
SCG> -----RHAAEVRRNKELQVELSG--------------
SCL> -----LHAAEVRRNKEQREEMSG--------------
STA> -----KHAEEVRKNKELKEEASR--------------
RB3> -----KHIEEVRKNKESKDPADETEAD----------
SYN> TNVGGAVVTGVTAVAQKTVEGAGSIAAATGFVKKDQL
               *    :
```

**Supplementary Figure S8**

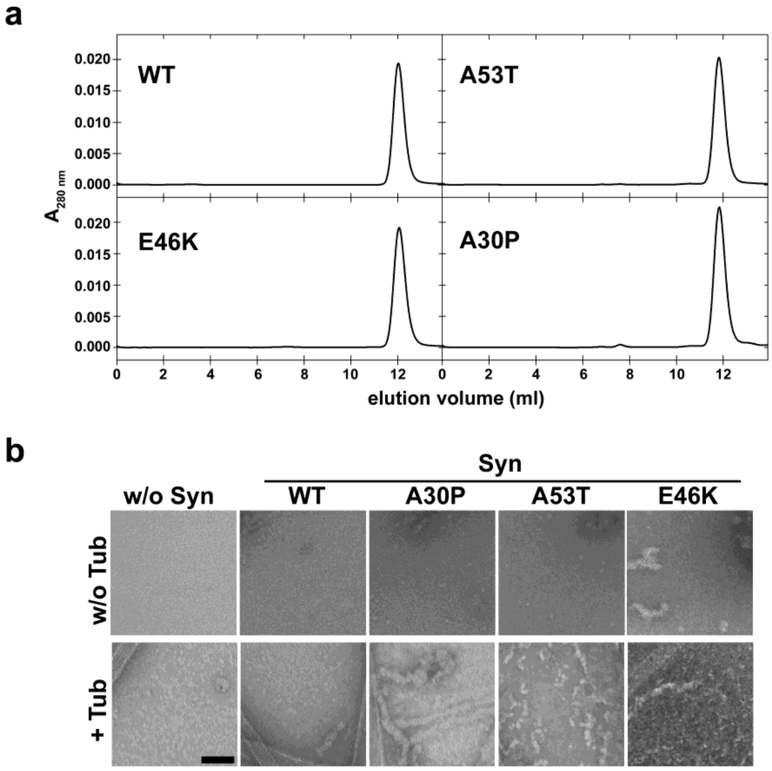

Supplement: Supplementary Information [file srep33289-s1.pdf]
